# Cerebrovascular Dysfunction in Atrial Fibrillation

**DOI:** 10.3389/fphys.2020.01066

**Published:** 2020-09-09

**Authors:** Rehan T. Junejo, Gregory Y. H. Lip, James P. Fisher

**Affiliations:** ^1^Liverpool Centre for Cardiovascular Science, Liverpool Heart and Chest Hospital, University of Liverpool, Liverpool, United Kingdom; ^2^Department of Physiology, Faculty of Medical and Health Sciences, The University of Auckland, Auckland, New Zealand

**Keywords:** atrial fibrillation, cerebral blood flow, carbon dioxide, hypertension, cerebral autoregulation, neurovascular coupling

## Abstract

It is now well established that besides being the most common sustained arrhythmia, atrial fibrillation (AF) is a major healthcare burden. Risk of debilitating stroke is increased in AF patients, but even in the absence of stroke, this population is at heightened risk of cognitive decline, depression, and dementia. The reasons for this are complex, multifactorial, and incompletely understood. One potential contributing mechanism is cerebrovascular dysfunction. Cerebral blood flow is regulated by chemical, metabolic, autoregulatory, neurogenic, and systemic factors. The dysfunction in one or more of these mechanisms may contribute to the elevated risk of cognitive decline and cerebrovascular events in AF. This short review presents the evidence for diminished cerebral blood flow, cerebrovascular carbon dioxide reactivity (i.e., cerebrovascular vasodilatory reserve), cerebral autoregulation, and neurovascular coupling in AF patients when compared to control participants in sinus rhythm. Further work is needed to understand the physiological mechanisms underpinning these observations and their clinical significance in atrial fibrillation patients.

## Introduction

Atrial fibrillation (AF) is the most common sustained cardiac arrhythmia characterized by an irregularly irregular cardiac output that leads to disrupted peripheral blood flow kinetics. Recognized as a major healthcare burden (Ball et al., 2013; Chugh et al., 2014), incidence and prevalence of AF is increasing in part due to the aging global population, better management of acute myocardial infarcts, and increasing occurrence of obesity and obstructive sleep apnoea (Wolf et al., 1996; Lane et al., 2017). The lifetime risk of developing AF in individuals aged ≥55 is currently reported as being 22 to 48% depending on the presence of risk factors (Heeringa et al., 2006; Weng et al., 2018).

AF is often accompanied by structural heart disease, vascular endothelial damage/dysfunction (Conway et al., 2003; Freestone et al., 2008), and abnormal blood constituents (Pourtau et al., 2017), which confer a prothrombotic hypercoagulable state. The risk of stroke is increased 5-fold in AF (Wolf et al., 1991) with cardioembolic events often being more severe, substantially increasing the risk of morbidity and mortality (Lin et al., 1996). However, AF patients, even if anticoagulated and with no clinical history of overt embolic ischemic stroke, present a heightened risk of cognitive decline, dementia, and depression (Bellomo et al., 2012; Marzona et al., 2012; Diener et al., 2019). This perhaps reflects silent infarcts (Conen et al., 2019), hypertension (Kim et al., 2020), systolic heart failure (Lee et al., 2019), hypercholesterolemia (Chao et al., 2015), and sleep apnoea (Leng et al., 2017), conditions that are individually associated with AF and cognitive impairment, necessitating holistic management of AF patients (Dagres et al., 2018). Nonetheless, one largely unexplored mechanism potentially contributing to the severe cerebrovascular events and cognitive dysfunction in AF patients is cerebrovascular dysfunction.

Cerebral blood flow is guided by the careful interplay of chemical, metabolic, autoregulatory, neurogenic, and systemic factors. The dysfunction in one or more of these mechanisms may contribute to the adverse cerebral events associated with AF. This mini-review will present evidence that AF modifies these aspects of cerebral blood flow regulation and the potential underlying mechanisms will be briefly discussed.

## Cerebral Blood Flow

Compromised cerebral perfusion may increase the risk of white matter damage and lower cognition (Thome et al., 1996; Jefferson et al., 2015). Lavy et al. (1980) and Gardarsdottir et al. (2017) documented a ~13% reduction in cerebral blood flow and cerebral perfusion in AF patients. Similarly, Junejo et al. (2019b) observed that cerebral perfusion, assessed using transcranial Doppler ultrasound measures of middle cerebral artery blood velocity (MVC Vm), was ~16% lower in AF patients [*n* = 31, 69 (64,72) years, median (interquartile range); 51.0 (12.9) cm s^−1^, mean (standard deviation)] when compared to healthy controls in sinus rhythm [*n* = 30, 69 (66,73) years; 60.9 (12.9) cm s^−1^; *p* < 0.01]. To assess AF irrespective of cardiac rhythm, comparisons were also made of AF patients diagnosed with paroxysmal (transient episodes that resolve spontaneously within 48 h) vs. persistent (untreated episodes last longer than 7 days) AF. Notably, fibrillating patients (55% of the total AF patients) exhibit a lower cerebral perfusion [44.4 (10.9) cm s^−1^] than non-fibrillating AF patients [59.2 (10.5) cm s^−1^; *p* < 0.01; Junejo et al., 2019b]. This supports the contention that the decreased cerebral blood flow in AF is driven by cardiac rhythm *per se*. A potential limitation of Junejo et al. (2019b) is that cerebral perfusion was assessed with transcranial Doppler ultrasound, which is limited to quantifying blood velocity but not blood flow, although a good correlation has been reported between transcranial Doppler ultrasound measures of velocity and cerebral blood flow (Clark et al., 1996; Poeppel et al., 2007). Similar findings regarding cerebral perfusion in fibrillating and non-fibrillating AF patients have been documented with phase-contrast MRI in a cross-sectional study (Gardarsdottir et al., 2017). Moreover, longitudinal studies with Xenon inhalation, single photon emission CT, arterial spin labeling, and phase-contrast MRI, where AF patients have undergone restoration of sinus rhythm, also demonstrate increases in global and regional cerebral perfusion (Petersen et al., 1989; Efimova et al., 2012; Gardarsdottir et al., 2019).

## Cerebral Carbon Dioxide Reactivity

The cerebral vasculature is very sensitive to changes in partial pressure of arterial carbon dioxide (CO_2_), with hypercapnia profoundly increasing cerebral blood flow and hypocapnia evoking cerebral vasoconstriction (Kety and Schmidt, 1946, 1948). Impaired cerebrovascular reactivity to CO_2_ (CVR_CO2_), indicative of an attenuated cerebrovascular reserve, is recognized as an independent predictor of ischemic stroke (Silvestrini et al., 2000; Markus and Cullinane, 2001) and cardiovascular mortality (Portegies et al., 2014). A poor CVR_CO2_ may increase the risk of severe ischemic stroke and delay functional recovery in AF patients.

**Figure 1:**
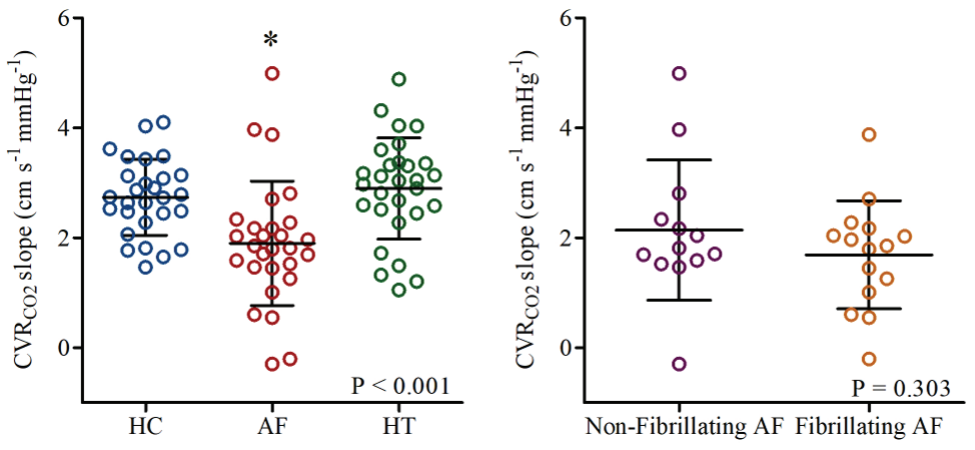
Cerebrovascular carbon dioxide reactivity (CVR_CO2_ slope) in healthy controls (HC: blue), patients with atrial fibrillation (AF: red), and hypertension (HT: green). Responses in AF-sub-groups (non-fibrillating and fibrillating AF patients) are shown on the right. Horizontal bars show mean and standard deviation (SD) for each group. ^*^p < 0.05 vs. HC and HT. Reproduced from Junejo et al. (2019b; open access article – Elsevier figure reuse license: 4825301232885; license date: May 10, 2020).

Junejo et al. (2019b) investigated whether CVR_CO2_ is impaired in AF patients [*n* = 31, 69 (64,72) years]. CVR_CO2_ was assessed using the slope of MCA Vm (transcranial Doppler ultrasonography) vs. partial pressure of end-tidal CO_2_ (P_ET_CO_2_; capnograph) by two 4-min step-increases in inspired CO_2_ fraction (4 and 7% CO_2_, respectively, ~21% oxygen and nitrogen balanced, open-circuit two-way valve method). Strikingly, CVR_CO2_ was ~31% lower in AF patients [1.90 (1.13) cm s^−1^ mmHg^−1^] compared to healthy [*n* = 30, 69 (66,73) years; 2.73 (0.69) cm s^−1^ mmHg^−1^; *p* < 0.001; Figure 1]. Given the potentially confounding effects of medications and comorbidities, comparisons were also made between AF patients and primary hypertension patients in sinus rhythm [*n* = 31, 68 (65,72) years] as a “disease” control group (Junejo et al., 2019b). CVR_CO2_ was documented to be ~34% lower in AF patients compared to hypertension patients [2.90 (0.92) cm s^−1^ mmHg^−1^; Figure 1; Junejo et al., 2019b]. The rationale for studying patients with hypertension was that hypertension heralds a 40–50% excess risk of developing AF (Benjamin et al., 1994), and is the most common coexisting cardiovascular disease in AF with prevalence ranging from 20–80% in patients diagnosed with AF (Heeringa et al., 2006; Miyasaka et al., 2006; Nabauer et al., 2009; Le Heuzey et al., 2010; Weng et al., 2018). To further control for comorbidities, all participants were free from left ventricular systolic dysfunction, valvular heart disease, history of myocardial infarction, stroke, secondary hypertension, insulin-dependent diabetes, malignancy, or uncontrolled thyroid disorders (Junejo et al., 2019b).

Whether the poor CVR_CO2_ identified in AF patients is primarily the result of cardiac rhythm *per se*, or is the result of damage caused by AF, is a key issue. One potential explanation for these findings is that a poor cerebral perfusion, secondary to the arrhythmia, may lead to a cerebral vasodilation that reduces the cerebral vasodilatory reserve (Aaslid et al., 1989). Indeed, as described above, AF patients exhibited a reduced MCA Vm particularly when fibrillating. However, Junejo et al. (2019b) observed that the attenuation in vasodilatory reserve of AF patients was unaffected by cardiac rhythm with no differences being observed between fibrillating [1.69 (0.98) cm s^−1^ mmHg^−1^] and non-fibrillating AF [2.14 (1.28) cm s^−1^ mmHg^−1^; *p* = 0.707] patients (Figure 1). Thus, suggesting that cerebrovascular dysfunction and specifically attenuated CVR_CO2_ in AF patients are independent of the cardiac rhythm and baseline cerebral perfusion *per se*.

Evidence of age‐ and hypertension-associated decline in CVR_CO2_ exists (Lipsitz et al., 2000; Walsh et al., 2009; Miller et al., 2019). However, findings of attenuated CVR_CO2_ in AF patients are novel and warrant further investigations. A potential explanation for the blunted CVR_CO2_ in AF patients is endothelial damage/dysfunction. AF evokes a turbulent blood flow pattern, loss of shear stress (Frangos et al., 1985; Noris et al., 1995) and oxidative stress (Sovari and Dudley, 2012), which collectively decrease the bioavailability of nitric oxide (NO), and arachidonic-acid derived vasodilators. Further, in AF, an attenuated brachial artery flow mediated dilatation response (FMD) (Freestone et al., 2008) and raised plasma von Willlebrand concentrations (Conway et al., 2003; Freestone et al., 2008), a factor related to adverse cardiovascular outcomes (Conway et al., 2003; Lip et al., 2006), have been identified and indicate endothelial damage/dysfunction. Both NO and arachidonic-acid derivatives are important in controlling cerebral blood flow during hypercapnia (Schmetterer et al., 1997; Kastrup et al., 1999). Indeed, administration of a NO-donor improves CVR_CO2_ in patients at risk of cardiovascular disease (Zimmermann and Haberl, 2003). Therefore, reduced production and bioavailability of endothelium-dependent vasoactive agents may underpin observations of reduced CVR_CO2_ in AF.

Debate surrounds the optimal method of assessing CVR_CO2_, and despite the wide use of fixed gas fractions and their relative ease to administer, they have received some criticism (Fierstra et al., 2013; Fisher, 2016). Junejo et al. (2019b) reported that MCA Vm and CVR_CO2_ showed good between-day test-retest reliability, nonetheless issues regarding between-subject and inter-operator variability remain. P_ET_CO_2_ is commonly used as a surrogate of arterial CO_2_ for measuring CVR_CO2_. Despite strong linear correlation between P_ET_CO_2_ and partial pressure of arterial CO_2_ (Peebles et al., 2007; McSwain et al., 2010), arterial CO_2_ concentrations can vary between participants and are dependent on multiple variables (e.g., metabolic state of individuals at the time of testing and alveolar ventilation variability), and P_ET_CO_2_ may underestimate arterial CO_2_ (Robbins et al., 1990; Delerme et al., 2010). Computerized sequential gas delivery offers fairly accurate estimates of arterial CO_2_ (Ito et al., 2008) and subsequently better estimates of CVR_CO2_. However, despite their advantages, financial setup costs and operator expertise (Fisher, 2016) have limited their widespread use. To date, only unidirectional (hypercapnic) cross-sectional comparisons of CVR_CO2_ using fixed gas fractions in AF patients either fibrillating or non-fibrillating have been made (Junejo et al., 2019b). Further, the impact of AF burden on progression of cerebrovascular dysfunction is currently not known. Longitudinal investigations of cerebral blood flow and tissue oxygenation/metabolism, both before and after restoration of sinus rhythm, warrant undertaking in combination with advanced imaging modalities.

## Cerebral Autoregulation

The cerebral vasculature possesses intrinsic mechanisms that maintain adequate perfusion despite fluctuations in blood pressure (i.e., cerebral autoregulation), thereby mitigating the risk of ischemia or hemorrhage by preventing under‐ or over-perfusion, respectively. Cerebral autoregulation functions as a high-pass filter whereby slower changes in perfusion pressure (>0.02 Hz) appear to pass unhindered, but more rapid pressure oscillation (<0.02 Hz) are dampened more effectively (Diehl et al., 1998).

Junejo et al. (2019a) assessed cerebral autoregulation in AF patients [*n* = 30, 69 (63,72) years], primary hypertensives [*n* = 29, 68 (65,72) years], and healthy controls [*n* = 24, 68 (66,70) years]. Cerebral autoregulation was determined using transfer-function analysis of the MCA Vm and blood pressure (finger photoplethysmography) responses to repeated squat-to-stand maneuver. AF patients exhibited greater changes in MCA Vm for a given change in blood pressure [gain normalized to baseline; 1.46 (1.16–2.16)% mmHg^−1^] compared to hypertensives [1.13 (1.00–1.45)% mmHg^−1^] and healthy controls [1.12 (0.99–1.37)% mmHg^−1^; *p* < 0.01], revealing impaired autoregulation. However, unexpectedly, sub-group comparison between AF patients showed that fibrillating AF patients (53% of total) were better able to delay blood pressure oscillations from transmitting into brain blood flow [phase; 0.63 (0.25) radians] compared to non-fibrillating AF patients [0.35 (0.17) radians; *p* < 0.01], and more effective at damping blood pressure driven changes to absolute measures of cerebral perfusion [absolute gain; 0.64 (0.22) vs. 0.92 (0.37) cm s^−1^ mmHg^−1^, respectively; *p* = 0.02]. However importantly, normalized gain failed to show any group differences between fibrillating [1.39 (1.11–1.80)% mmHg^−1^] and non-fibrillating [1.56 (1.30–2.23)% mmHg^−1^; *p* = 0.29] patients.

Impaired autoregulation may result from a number of interactive mechanisms, including mechanosensitive myogenic ion channels (Davis et al., 1992; Tan et al., 2013), neurogenic/autonomic influences (Hamner et al., 2012; Hamner and Tan, 2014), metabolic influences (Panerai et al., 1999), and NO (White et al., 2000). More specifically, autonomic disturbances (Chen et al., 2014), endothelial damage/dysfunction (Conway et al., 2003; Freestone et al., 2008), and diminished bioavailability of endothelial vasodilators (Minamino et al., 1997) in AF could contribute to autoregulatory dysfunction of AF. Further, the reduced cerebral blood flow and vasodilatory reserve observed in AF patients (Junejo et al., 2019b) may also impair cerebral autoregulation. Further investigations into the mechanisms of impaired autoregulation of AF are warranted.

Collectively, these observations suggest that cerebral vasculature in AF patients is less able to buffer blood pressure driven fluctuations in brain blood flow (i.e., cerebral autoregulation is impaired) in comparison with primary hypertensives and healthy individuals in sinus rhythm. The apparently conflicting finding of improved absolute gain in fibrillating AF patients may just reflect fibrillation itself, rather than an improvement in cerebrovascular health during fibrillation.

## Neurovascular Coupling

The regional metabolic needs of neuronal activation share a close spatial and temporal fidelity with local blood flow, as such ensuring commensurate functional perfusion within the brain (Phillips et al., 2016). This phenomenon is commonly referred to as neurovascular coupling. An impaired neurovascular coupling, indicative of cerebrovascular dysfunction, has been reported post-stroke, associated with cognitive decline, and linked to endothelial dysfunction (Girouard and Iadecola, 2006; Graves and Baker, 2020).

Junejo et al. (2019a,c) investigated whether neurovascular coupling is blunted in AF patients [*n* = 12, 71 (66,72) years] compared to primary hypertensives [*n* = 13, 66 (65,69) years] and healthy controls [*n* = 12, 69 (57,70) years]. Beat-to-beat posterior cerebral artery (PCA), MCA Vm (temporal transcranial Doppler ultrasonography), and vascular conductance (calculated as Vm/mean blood pressure) responses to repeated visual-stimuli (30 s eyes-open, 30 s eyes-closed for 5 min) were spline interpolated and then averages and percentage changes calculated (Phillips et al., 2016). This allowed account of changes to blood velocity and vascular diameter along with any inadvertent blood pressure fluctuations during testing. Neurovascular coupling was defined as the visually evoked increase in PCA conductance, since the PCA supplies the visual cortex.

**Figure 2:**
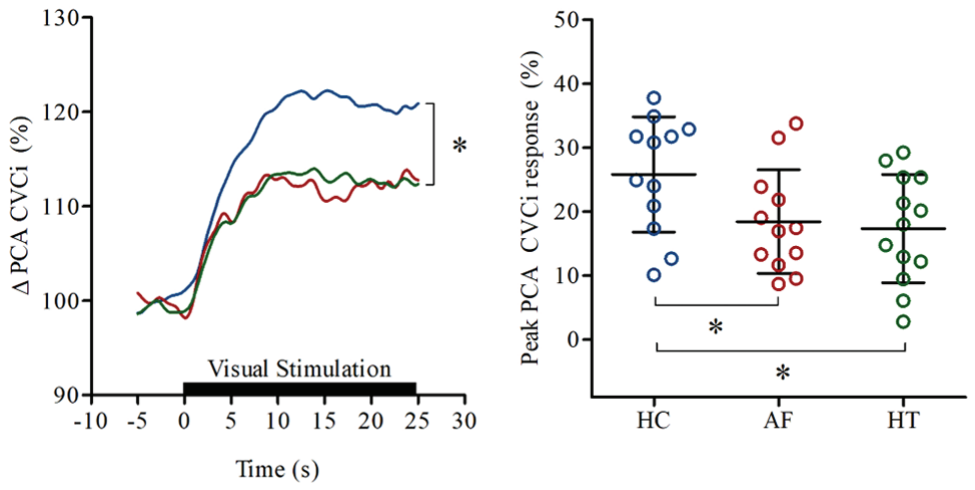
Increase in PCA perfusion in response to neurovascular coupling observed in healthy controls (HC: blue), patients with atrial fibrillation (AF: red), and hypertension (HT: green). Lines on the **left panel** represent the mean responses; black bar indicates where eyes of the participants were open. **Right panel** shows the (%) peak posterior cerebral artery (PCA) conductance (CVC_i_) responses of individuals. Horizontal bars show mean and SD values for each group. ^*^p < 0.05 vs. HC. Modified from Junejo et al. (2019a; open access article published under CC-BY 4.0 license).

A blunted peak PCA conductance was observed in AF [18 (8)%] and hypertensive patients [17 (8)%] compared to healthy controls [26 (9)%; *p* < 0.05], indicative of blunted neurovascular coupling in people with either AF or hypertension, relative to control participants (Figure 2). However, the change in MCA conductance in AF patients [17 (6)%] was greater than hypertensives [10 (4)%; *p* < 0.05], suggesting non-specific neurovascular engagement of cerebral areas in AF patients. To explore this issue further, visual stimulation related task-specificity was calculated as the difference between the PCA and MCA conductance responses. This analysis revealed that the neurovascular coupling response was near-completely abolished in AF patients [1.0 (7.5)%] compared to hypertensives [6.6 (9.4)%] and healthy controls [12.9 (9.2)%; *p* < 0.01].

These results indicate reduced neurovascular coupling responses in AF patients; however, the underlying mechanisms remain unclear. Neurovascular coupling is mediated by a complex array of feed-forward and feedback mechanisms, (e.g., hydrogen, potassium, adenosine, prostaglandins, NO, acetylcholine, glutamate, and dopamine; Girouard and Iadecola, 2006; Phillips et al., 2016). Further, recent evidence from animal models also suggests an active role of nicotinamide mononucleotide in neurovascular coupling response (Tarantini et al., 2019a,b). Evidence exists for age‐ and hypertension-associated decline in neurovascular coupling response (Girouard and Iadecola, 2006; Lipecz et al., 2019), alongside some conflicting reports (Stefanidis et al., 2019). However, mechanistic studies in humans are limited and it remains to be investigated whether the attenuated neurovascular coupling responses reported in AF patients (Junejo et al., 2019a,c) reflect neurodegenerative blunting or disrupted coupling between neurons and vasculature. Moreover, to our knowledge, to date, neurovascular coupling has only been assessed in AF patients using visual stimulation (i.e., reading) and whether this diminished response persists during other stimuli (e.g., finger tapping) is unknown.

## Mitigation Strategies

Improvements in cerebral perfusion and cognitive function have been observed with cardiac rhythm control following pharmacological (Damanti et al., 2018), cardioversion (Petersen et al., 1989), ablation, and pacemaker treatments (Efimova et al., 2012). Besides the improvements in ventricular filling and systolic function, rhythm control strategies offer improvements in endothelial function (Noris et al., 1995; Topper et al., 1996; Skalidis et al., 2007). Nonetheless, it is important that any rhythm control strategies employed in AF patients to improve cerebral and systemic perfusion, and vascular/endothelial health are carried out alongside parallel and continued antithrombotic therapy. Indeed, the heightened risk of ischemic strokes continues even after cardiac arrhythmia correction in AF patients (Lip, 1995; Thibault et al., 2004).

Increased concentrations of circulating inflammatory markers observed in AF suggest their contribution to endothelial damage and prothrombotic platelet activation (Patel et al., 2010; Guo et al., 2012). However, in a feedback loop, coagulation can also influence inflammation and encourage vascular dysfunction (Levi et al., 2004; Esmon, 2005). Thus, it is possible that besides the reduction in procoagulants and subsequent reduction in stroke risk (Members et al., 2012), factor Xa, thrombin, and/or vitamin K antagonists also help attenuate cerebrovascular and peripheral vascular dysfunction in AF.

Exercise training, whether endurance (Lautenschlager et al., 2008; Green and Smith, 2017) or resistance (Cassilhas et al., 2007), oral antioxidant (Wray et al., 2012) and nitrite (Lara et al., 2016) supplementation, ischemic preconditioning (Jones et al., 2014), and heat therapy (Brunt et al., 2016) have all been associated with improved cardiovascular and/or cerebrovascular health. Their employment to improve cerebrovascular function and mitigate the risk of cognitive decline in AF patients remains a valid proposition; however, objective evidence for their effectiveness in AF remains lacking.

## Conclusion

AF is associated with an increased stroke risk, and even anticoagulated AF patients are at an increased risk of cognitive decline, depression, and dementia. Emerging evidence suggests impaired cerebral vasodilatory reserve, autoregulation, and neurovascular coupling in AF patients compared to “disease” (primary hypertension) controls and healthy controls in sinus rhythm. These findings may be important in explaining the severity of ischemic strokes, morbidity, and mortality risk from such events, cognitive decline, and cerebral dysfunction in AF. Further cross-sectional and longitudinal studies are needed to better understand the pathophysiological underpinnings and clinical significance of these findings.

## Author Contributions

RJ drafted the manuscript which was critically revised by GL and JF. All authors contributed to the article and approved the submitted version.

### Conflict of Interest

GL is a consultant for Bayer/Janssen, BMS/Pfizer, Medtronic, Boehringer Ingelheim, Novartis, Verseon, and Daiichi-Sankyo and speaker for Bayer, BMS/Pfizer, Medtronic, Boehringer Ingelheim, and DaiichiSankyo. No fees are directly received personally. JF has received funding from BMS/Pfizer for an investigator-led and competitively reviewed research project. RJ was previously employed by University of Birmingham as a research fellow on BMS/Pfizer funded, investigator-led and competitively reviewed research project which he managed. This required regular contact with funders for continued project grant support. No fees/funds were directly received personally.
